# Supplementary material for: Are carbon emissions trading and green financial instruments synergistic? -Comprehensive quantitative research based on content analysis
Source: PLoS One. 2024 Mar 7;19(3):e0298601. doi: 10.1371/journal.pone.0298601 (PMC10919601; doi:10.1371/journal.pone.0298601)
Supplement: S1 Data — (ZIP) [file pone.0298601.s001.zip › Data/地级市Z_F工作报告.zip!/╜¡╬≈/╛░╡┬╒≥╩╨/└┤╫╘╣1⁄2╓┌║┼í╛┬φ┐╦╩2╛▌═°í┐_2011.docx]

各位代表：现在，我代表市人民政府向大会作工作报告，同时就《景德镇市国民经济和社会发展第十二个五年规划纲要（草案）》作说明，请予审议，并请各位市政协委员和列席会议的同志提出意见。

一、“十一五”时期工作回顾

“十一五”时期，在省委、省政府和市委的正确领导下，全市广大干部群众团结协作，真抓实干，战胜了历史罕见的低温雨雪冰冻灾害和特大洪涝灾害，全面完成了“十一五”规划确定的主要目标和任务，全市经济社会发展取得了新的成就。五年来，我们主要做了以下几项工作。

（一）全面推进经济持续较快增长着力巩固“三农”基础。

始终把促进粮食稳产增产工作作为重中之重，粮食产量由2005年的49.2万吨增加到57.5万吨，实现“七连增”；不断增加农业基础设施投入，全市农业综合开发投入累计完成2.7亿元，比“十五”增加1.7亿元，水利建设投入6.6亿元，增加5.3亿元，除险加固病险水库192座，乐平市和浮梁县被列入全国小型农田基本建设重点县；进一步提高农业产业化经营水平，规模以上农业产业化龙头企业销售收入34亿元，比“十一五”期末增长65.9%；扎实推进社会主义新农村建设，累计投入资金4.9亿元，建设新农村试点村764个，初步形成了“一村一品”的产业格局。

加快推进工业化进程。工业生产总量迈上新台阶，规模以上工业增加值达160亿元，比“十五”期末净增111亿元，规模以上工业企业达437户，五年新增188户；陶瓷产业实现四大突破，总产值达160亿元，出口3亿美元，上缴税收2.66亿元，陶瓷规模企业数量超过百家；工业园区强劲发展，三大园区主营业务收入370亿元，是“十五”期末的9.5倍，占全市工业的比重达到46.5%，比“十五”期末提高28.4个百分点；市高新区升格为国家级高新技术产业开发区，陶瓷工业园被科技部列为国家火炬计划基地；节能减排成效显著，万元生产总值能耗从1.24吨标准煤下降为0.95吨标准煤，化学需氧量控制在2.17万吨以下，二氧化硫排放量控制在4.01万吨以下；国企改革取得重大突破，国有工业企业和六大系统国有非工业企业改革基本完成；金融业改革发展成效显著，存贷款规模大幅度增长，市农村商业银行、市商业银行、浮梁县农商村镇银行挂牌开业，成功引进九江银行，黑猫股份实现上市再融资，世龙实业进入上市辅导期。

积极发展开放型经济。招商引资的数量和质量快速提升，2010年，全市引进3000万元以上项目145个，实际进资239.7亿元，比“十五”期末增长458.6%;新审批外资项目23个，实际利用外资1.2亿美元，增长137.9%;实际引进内资增速连续四年进入全省前三名，并在2009年首次获得全省开放型经济发展综合奖；工业项目引进成效斐然，光伏、陶瓷、汽车零部件、家电和LED照明等一大批重大产业项目落户，爱地那非和可利霉素项目将填补我省国家一类新药生产的空白；外贸出口创历史新高，五年累计出口17.6亿美元，比“十五”增长五倍多，2010年达7.8亿美元，是2005年8.8倍；景德镇瓷博会已成为集陶瓷产品展示、贸易投资和文化交流于一体，具有较大国际影响的国家级博览会。

全力推进重大基础设施建设。能源供应体系建设取得重大突破，变电站数量和容量、输电线路条次和长度实现翻番，基本上再造了一个景德镇电网，景德镇电厂“上大压小”扩建工程1号机组并网发电，创造了全国同类型机组建设工期最短纪录，天然气利用工程全部完工；交通建设快速发展，“十一五”交通基础设施建设投入62.2亿元，建成高速公路超过121公里，改造国、省道主干线155.8公里，新建、改造农村公路1376.3公里，提前两年实现了行政村通沥青（水泥）路；景德镇机场新航站楼投入使用；皖赣铁路电气化改造、九景衢铁路新建项目获得国家批准。

（二）着力开展宜居城市建设

努力健全城市规划体系。修编完善的《景德镇市城市总体规划(2010-2030)》已经省政府原则同意；编制了《景德镇市历史文化名城保护规划》等中长期规划，完成了《景东大道两侧控制性详细规划》等45项详规，中心城区详规覆盖率由“十五”期末的64%提高到90%。不断完善城市功能。城区面积由“十五’’期末不足40平方公里扩大到82.5平方公里。白鹭大桥、民窑遗址博物馆等工程相继建成。景东大道、景德大道东段等城市主干线全面贯通。市妇幼保健院和光明电影院、中国陶瓷博物馆等公共建筑的主体工程完工。投资近10亿元保护和改造了戴家弄、斗富弄、新村东路水淹房等7大片老城区。建设了九大公园、五大广场、一江两岸景观带，新增公园和广场的绿地面积1万多亩。有效保护生态环境。全市森林覆盖率由“十五”期末的58.9%提高到65.4%，建成区绿化覆盖率由42%提高到52.1%。完成了西瓜洲城市污水处理工程，第二城市污水处理厂建成并投入使用，生活垃圾无害化处理率由92%提高到99.3%。多次获得全省城市环境定量考评第一名。广泛开展城市创建活动。在全省率先建成数字化城市管理中心，获得创建全国文明城市工作先进城市、国家园林城市、中国优秀旅游城市：中国人居环境（范例）奖、全国绿化模范城市等荣誉称号。积极争取成为国家各类改革试点城市。我市已成为国家第二批资源枯竭型城市、全国创新型试点城市、全国首批服务业综合改革试点城市、全国第三批节水型城市建设试点和全国第二批再生资源回收利用试点城市。

（三）大力促进文化旅游产业快速发展

推进文化创意产业发展。制定了《陶瓷文化艺术创意产业发展规划》，建立了江西省第一家文化创意产业基地一雕塑瓷厂陶瓷文化产业园，开工建设了国际陶瓷文化创意产业园，启动了景德镇御窑遗址公园保护与建设。曙光瓷厂、建国瓷厂、红店街等陶瓷文化创意产业项目建设初见成效。景德镇古窑复烧入选吉尼斯纪录。海畅集团、陶瓷历史文化博览区被列为全国文化产业示范基地。加大非物质文化遗产保护。非物质文化遗产列入国家级名录2项、省级10项。非物质文化遗产代表性传承人列入国家级4人、省级11人。陶瓷文化对外交流进一步扩大。成功举办海峡两岸陶瓷精品展、“辉煌60年一景德镇陶瓷成就成果展”、“中国瓷都景德镇一首届上海陶瓷成就展”等活动。旅游产业快速发展。2010年，全市旅游接待总人数由2005年的585.6万人次上升到1324.6万人次；旅游总收入由21亿元上升到66.6亿元，占GDP的比重由10.9%上升到14.4%。

（四）统筹推进社会事业协调进步扎实有效实施民生工程。

全面完成省政府下达的民生工程任务，“十一五”期间，累计城镇新增就业13.3万人，城镇就业率达96.5%，转移农村劳动力14.1万人；累计建设经济适用房36.9万平方米、廉租房24.5万平方米；“十一五”期末，城镇职工参加基本养老保险达到34.4万人，其中农民工3.4万人，新型农村合作医疗参合率达到99.1%；不断提高低保标准，城市和农村低保补差水平分别达到每月180.4元和75元，农村五保户集中供养率达到80.2%。协调推进社会各项事业。教育事业取得长足发展，全市公民人均受教育年限居全省领先水平，“两基”工作通过国家验收，新增省重点中学和省重点建设中学各3所，高等教育办学规模进一步扩大，职业教育、特殊教育、民办教育和老年教育同步发展；科技进步不断加快，部省共建景德镇国家陶瓷科技城和国家知识产权试点工作深入推进，荣获国家科技进步二等奖1项，省科技进步一等奖2项；全民健身活动遍及城乡，竞技体育取得丰硕成果，吴静钰、金紫薇在北京奥运会和多哈、广州亚运会连夺金牌，为瓷都赢得声誉；公共卫生体系不断完善，社区卫生服务实现全覆盖，全市共建设三级甲等医院4所、二级甲等医院3所，“光明微笑”工程获得表彰；食品药品安全保障体系不断完善；连续四次成为全国双拥模范城；同时，人事编制、供销、质监、航务、气象、水文、民族、宗教、外事侨务、新闻出版、对台、老龄、慈善、妇女儿童、残联、海关、商检、人防、档案、地方志、工商、通讯、保险、石油、盐业等其他各项事业也都取得了新的进步。保持社会和谐稳定。社会治安防控体系建设成效显著，治安好转率连续三年全省第一，获得了“全国社会治安综合治理优秀城市”荣誉称号，群众安全感进一步增强；维稳工作机制进一步健全，率先在全省设立了医患纠纷调处中心，建立完善了矛盾纠纷调处机制；依法打击非法小煤窑成效显著，连续4年实现了“零开采、零死亡”，全市连续8年未发生一起死亡10人以上的重特大安全生产事故。

（五）不断提高依法行政水平

政府及其职能部门自觉接受人大及其常委会的法律监督、政协的民主监督，办理了人大代表意见建议425件、政协提案662件，办结率名列全省前茅；“五五”普法工作取得优异成绩，依法治市进程不断加快；大力推进行政审批制度改革，“两集中、两到位”工作取得初步成效；深入推进政务公开，网上审批和电子监察工作走在全省前列；认真开展机关效能建设和创业服务年活动，连续两年绩效考核荣获全省第一名；进一步加强廉政建设和反腐败工作，抓好治理商业贿赂工作，积极推行风险岗位廉能管理，扎实开展民生领域三个突出问题和扩大内需投资项目及工程建设领域突出问题等专项治理；有重点、有成效地开展了各种专项审计工作，审计监督机制逐步健全。

各位代表，经过五年努力，2010年全市生产总值由2005年的193.1亿元，增加到461.5亿元，年均增长14.9%；财政总收入由14.7亿元增加到53.1亿元，年均增长29.3%；全社会固定资产投资由89亿元增加到439.6亿元，年均增长37.9%；社会消费品零售总额由58.3亿元增加到140.4亿元，年均增长19.5%；城镇居民人均可支配收入由8779元增加到16657元；农民人均纯收入由3589元增加到6521元；人口自然增长率下降到7.73‰。过去的五年，是我市发展提速、后劲增强的五年，是城市宜居、社会和谐的五年，也是民生改善、人民受益的五年。这五年我们基本建成了一座特色宜居的城市；这五年我们基本完成了陶瓷产业的解困；这五年我们的产业与城市转型迈出了坚实的步伐。

各位代表，能够出色地完成上述工作，是省委、省政府和市委正确领导的结果，是市人大依法监督和市政协积极参政议政的结果，是全市人民辛勤劳动的结果。在此，我代表市人民政府，向全市广大工人、农民、知识分子、干部和离退休老同志，向各民主党派、无党派、工商联、人民团体和社会各界人士，向驻市解放军、武警官兵和公安干警，向来景的投资者和外来劳动者，向所有参与、关心和支持瓷都发展的海内外友好人士表示衷心的感谢！在肯定工作成绩的同时，也应清醒地看到，我们还面临着许多困难和问题：经济规模和总量仍然较小，城市综合实力仍然较弱；结构调整、产业升级、城市转型的任务还很艰巨；公共产品供给与人民群众期待之间仍然存在差距，人民群众的生活水平还有待提高；政府自身建设有待于进一步加强等。我们将高度重视这些困难和问题，并在今后的工作中努力克服和解决。

二、“十二五”时期的主要任务

根据《中共景德镇市委关于制定全市国民经济和社会发展第十二个五年规划的建议》，市政府制定了《景德镇市国民经济和社会发展第十二个五年规划纲要（草案）》（以下简称《纲要（草案）》），提交本次大会审议。根据《纲要（草案）》，“十二五”时期全市经济社会发展的指导思想是：高举中国特色社会主义伟大旗帜，以邓小平理论和“三个代表”重要思想为指导，深入贯彻落实科学发展观，适应国内外形势新变化，顺应人民群众新期待，以科学发展为主题，以加快转变经济发展方式为主线，以改革开放和科技创新为动力，以保障和改善民生为根本，以鄱阳湖生态经济区建设为龙头，加速推进新型工业化、加速推进新型城镇化、加速推进农业农村现代化，着力完善基本公共服务体系，着力提高人民群众幸福指数，努力在科学发展、进位赶超、绿色崛起的进程中建设成为全省、全国乃至全世界具有特色和影响的战略性新兴产业基地、转型创新试验区域、生态文明宜居城市。《纲要（草案）》提出了“十二五”时期的主要目标。在经济发展方面，到2015年全市生产总值翻一番，总量突破1000亿元，年均增长14%以上，人均生产总值力争达到8000美元以上；财政总收入达到150亿元，年均增长确保18%、力争20%以上；全社会固定资产投资力争900亿元，年均增长20%;全社会消费品零售总额达到300亿元，年均增长16%；出口总额突破15.6亿美元，年均增长15%;产业结构进一步优化，三次产业比重调整为5：54:41;城镇化率达到60%以上。在生态环境建设方面，主要指标位居全省前列。全市监测断面水质达标率达到97%;城市空气优良率99.7%；万元GDP能源消耗持续稳定下降；二氧化硫排放量、化学需氧量排放量持续下降；城镇生活污水集中处理率、城镇生活垃圾无害化处理率均达到80%；森林覆盖率稳定在65%以上。在社会发展方面，人口自然增长率控制在8‰以内；城镇调查失业率得到有效控制，五年新增就业13万人；巩固提高“普九”成果；基本养老保险参保人数达到40万人，城镇基本医疗保险覆盖率提高到100%，新型农村合作医疗农民参合率保持在96%以上；城镇人均可支配收入和农民人均纯收入年均增长11%，分别达到28000元和11000元。《纲要（草案）》提出，“十二五”时期要重点抓好以下几个方面的工作。

第一，大力发展战略性新兴产业，加快资源型城市转型。要优化产业结构，发展战略性新兴产业，促进传统产业升级，提升工业园区发展水平；要以直升机、陶瓷和医药为重点，加快发展高新技术陶瓷及创意、航空、生物和新医药、光伏、清洁汽车及动力电池、LED半导体照明、现代农业和有机食品等八大战略性新兴产业；要加快服务业发展，积极推进国家服务业综合改革试点，一打造赣东北物流服务平台，推动金融服务业发展。

第二，继续夯实农业发展基础，建设社会主义新农村。要稳定粮食播种面积，严格保护耕地，加强以农田水利设施为基础的田间工程建设，建设一批旱涝保收高标准粮田，强化粮食生产扶持政策，培植粮食产业园；要积极发展现代农业，推进农业发展方式转变，调整优化农业结构；要加快社会主义新农村建设，科学规划乡镇村庄布局，加强农村基础设施建设，提升农村环境质量；要提高农民收入水平，拓宽农民增收渠道，积极引导农村富余劳动力有序转移就业，做好扶贫工作；要深化农村综合改革，坚持和完善农村基本经营制度，完善城乡平等的要素交换关系，深化农村信用社改革，创新农村土地管理制度。

第三，推动公共文化事业繁荣发展，打造“文化旅游强市”。要推动公共文化事业大繁荣，加大文化遗产保护力度，完善公共文化服务体系，开展群众性文化活动，繁荣文化市场，发展体育事业，加强精神文明建设；要大力发展陶瓷文化创意产业，建设创意经济城市，推进古陶瓷研究与发展；要加快旅游业发展，大力实施旅游景区提升工程，创新旅游营销方式，提高旅游服务功能。

第四，推进新型城镇化，建设鄱阳湖生态经济区中心城市。要科学规划中心城市建设，优化城市布局；壮大县域经济实力，鼓励发展各具特色的县域优势产业，力争“十二五”期末，各县（市、区）经济总量、固定资产投资、财政收入翻两番；推进城镇化进程，促进城乡一体化。

第五，完善重大基础设施，为经济发展提供有力支撑。要加快水利工程建设，提高防洪减灾救灾能力，建设安全可靠的水利保障体系；要对接环鄱阳湖城市群高速通道工程，推进全市新一轮铁路、公路建设，加强航道建设，构建高效便捷的综合交通运输体系；要强化对煤、电、油、气等重要生产要素的调度保障，建设清洁高效的能源保障体系；要加快信息网络建设，加快信息技术开发应用，建设覆盖城乡的信息网络体系。

第六，促进生态经济融合，提升生态文明水平。要加强能源资源集约利用，强化节能降耗，积极申报低碳试点城市；要加强环境保护，控制污染物排放；要大力发展循环经济，推广清洁生产方式，构建绿色消费模式，加强资源回收利用，完善政策技术支持；要建立健全生态保障体系，建设生态屏障，加强生态治理；要建立科学防灾减灾体系，提高应对气候变化能力。

第七，推进科教兴市战略，加快建设人力资源强市。要建设创新型城市，加强创新型企业建设，加强创新平台建设，打造优势科技创新团队，完善创新机制；要优先发展教育事业，大力促进教育公平，建立促进教育发展的体制机制；要实施人才强市战略，建设人才队伍，加强人才培养，改善发展环境。

第八，切实保障和改善民生，共享改革发展成果。要提高社会保障水平，实施更加积极的就业政策，完善社会保障体系，加快医疗卫生事业改革发展；要加强保障性住房建设，增加保障性住房供给；要强化公共安全保障能力，夯实安全生产基础，加强食品药品安全监管工作，加大公共安全投入；要推进和谐社会建设，创新社会管理机制，健全社会救助服务体系，全面做好人口工作；要加强民主和法制建设，构建和谐社会；要全面推进依法行政、公正廉洁执法。

第九，全面深化各项改革，激发发展活力。要深化行政管理体制改革，进一步转变政府职能，建设法治政府和服务型政府；大力推进投资体制、财税体制和国有企业的改革；扎实做好先行先试改革试点工作。

第十，发展开放型经济，加强对外合作。要提升开放型经济水平，加大招商引资力度，加快对外贸易转型升级；优化对外开放环境，拓展合作平台，提升服务能力。《纲要（草案）》最后提出，要完善实施机制，举全市之力，集全民之智，充分调动广大人民群众的积极性、主动性和创造性，确保实现“十二五”发展的宏伟蓝图。

三、2011年主要工作今年是实施“十二五”规划的开局之年，全市经济社会发展的主要目标是：全市生产总值增长14%；财政总收入、地方财政收入均增长18%；全社会固定资产投资增长22%；社会消费品零售总额增长18%;外贸出口增长15%；实际利用外资增长12%;实际引进内资增长25%;规模以上工业增加值增长20%；城镇居民人均可支配收入增长11%;农民人均纯收入增长11%;人口自然增长率控制在8%0以内；万元生产总值能耗下降3.5%。为实现上述目标，我们将重点抓好以下几项工作。

（一）突出农业增产、农民增收，扎实做好“三农”工作进一步稳定粮食生产。加大粮食和农业生产投入，继续开展保护基本农田、高产稳产农田建设和农业配套服务三大体系建设。抓好春耕备耕，大力实施“造地增粮富民”工程，净增耕地面积4000亩。加大中低产田改造力度，开展粮食高产创建活动，加快农业机械化进程，提高粮食综合生产能力。2011年，力争粮食播种面积稳定在138万亩，粮食总产57.5万吨。进一步抓好农田水利建设。扎实开展“农田水利基本建设年”活动，重点抓好水毁工程修复和农田水利基础设施建设。完成国家规划治理的34座病险水库和我市计划治理的374座小(2)型病险水库的应急除险加固任务。推进农田灌溉“润田工程”建设。加快南河湘湖段治理工程、牌楼圩农田防护工程建设。深入推进乐平市、浮梁县全国小型农田水利重点县建设。进一步提升农业产业化水平。围绕蔬菜、茶叶、油茶、养殖等特色产业发展农产品加工业，实现农产品多次增值。认真落实“菜蓝子，市长负责制，进一步加强城市农贸市场管理，严厉打击欺行霸市等不法行为。加快实施乐平市蔬菜种植加工和批发市场建设、浮梁县茶叶低改扩种和名优茶开发加工、10万头生猪养殖及加工、德宇白花木等产业化项目，加快建设有机蔬菜基地、有机茶叶基地和有机油茶基地。全市农业产业化经营销售收入力争突破45亿元，规模以上龙头企业超过85家，带动19万户农户增收，力争农民专业合作社发展到350个。加大农产品“农超对接”力度，积极开展名优特农产品展示展销洽谈活动，加快构建市县两级农产品质量安全检验监测网络。进一步加大社会主义新农村建设力度。要促进生产发展，抓好“一村一品”、“一村一图”，着力形成一批特色鲜明、类型多样、竞争力强的专业村；要抓好基础村的整治建设，强化农村环境保护，争取创建省级生态乡镇6个，省级生态村9个；要加快农村新社区建设，进一步健全村民自治机制，促进乡风文明和民主管理；要抓好205个新农村的试点建设，力争让更多农民群众“走平坦路、喝干净水、上卫生厕、住整洁房、用洁净能源、过文明生活”。

（二）培育壮大战略性新兴产业，推进新型工业化进程遵循“结构优化、技术先进、清洁安全、附加值高、吸纳就业”的原则，培育壮大高新技术陶瓷及创意、航空、生物和新医药、光伏、清洁汽车及动力电池、LED半导体照明、现代农业和有机食品等八大战略性新兴产业。壮大和提升陶瓷产业的总量和质量。今年力争完成陶瓷工业总产值192亿元，陶瓷出口3.3亿美元。

第一，优先发展高新技术陶瓷。加强陶瓷创新体系建设，加速高新技术陶瓷产业化、配套化、集群化；重点扶持远红外陶瓷、电子陶瓷、蜂窝陶瓷、光通讯陶瓷等高技术项目和低膨胀陶瓷、陶瓷薄板、陶瓷辊棒、纳米碳酸钙等新材料项目的发展。

第二，大力发展陶瓷文化创意产业。要支持和鼓励陶瓷企业、科研院所、高校和艺术家工作室大力开展陶瓷制品创意设计、陶瓷工艺美术设计、陶瓷产业软件设计；推进建设景德镇国际雕塑创意园、建国陶瓷文化创意园、红店街、三宝国际陶艺村等陶瓷文化创意项目；以瓷博会为平台，进一步扩大陶瓷文化创意产业的交流与合作；加快国际陶瓷艺术创意中心建设，为集聚国际高端陶瓷创意人才、开展高水平陶瓷创意活动，打造重要的原创设计和人才基地。

第三，加快陶瓷企业的改造。要依托部省共建景德镇国家陶瓷科技城，加快陶瓷的产业、科研、教育、贸易四大基地建设；要加大陶瓷企业技术改造力度，加快培育大型陶瓷龙头企业，积极推进企业上市；要加强陶瓷品牌建设，充分发挥现有国有陶瓷资源的作用，加快整合陶瓷科研贸易和十大瓷厂的力量，对陶瓷改制企业商标、商誉及专有技术等无形资产进行全面清理和统一管理，积极打造好、维护好、运用好景德镇陶瓷品牌，以品牌建设推动陶瓷企业核心竞争力和文化软实力的提升。第四，努力创造新一代陶瓷传世精品。要支持古典陶瓷研究与发展，充分利用我市丰富的陶瓷历史文化遗存，依托一批优秀陶瓷考古、研究和鉴定人才及非物质文化遗产传承人，逐步把景德镇建设成为古典陶瓷研究的国际中心、复制利用的高地、科技鉴定和人才培训的基地；要更加注重将古典陶瓷生产工艺和生产技术与现代时尚和世界潮流相结合，开发令人耳目一新的新一代陶瓷精品，在实现古典陶瓷复兴的基础上，创造出具有时代特征的新古典主义陶瓷。建设中国直升机研发制造基地。随着国家低空空域的逐步放开，航空产业将迎来跨越发展的黄金时期。我们一定要加快形成以研发为核心，总装为标志，集零部件配套、国际合作、培训维修为一体的直升机产业体系，建设有特色、有竞争力的中国直升机研发制造基地；要全力推动扩大和拓展民用直升机的研发平台、总装能力、国际合作、零部件配套、培训和维修能力；要建立高效管理体制，做好协调和服务工作；要制定更加宽松的扶持政策，优先安排用地指标，完善人才激励机制，实行财税优惠政策，努力促进中国直升机研发制造基地的建设和发展。壮大生物和新医药产业。要推动高科技生物制药企业加大新产品研发力度，提升企业核心竞争力；要为爱地那非和可利霉素的生产创造良好环境，提供优质服务，支持国家一类新药加快成为拳头产品；抓好阿莫西林等重大医药项目的竣工投产，促进医药产业成为战略性支柱产业；更加注重医药行业的污染防治工作，坚决不上污染青山绿水的医药项目。

（三）打造生态宜居特色瓷都，推进新型城镇化和城市建设

加强老城区保护。保护老城区具有保留历史、保护文化、保存特色、保有城市精神的重大意义。要按照统一规划、分步实施、先易后难的策略，把老城区建设成为原汁原味原生态、宜居宜游宜就业的瓷风瓷韵“景德镇’’，要突出保护古旧里弄中的文物、历史和建筑风貌，保留传统瓷业生产遗迹，传承镇派里弄、建筑、陶瓷文化和人文精神、民间习俗；要重点以御窑为核心，以中华路、中山路为轴线，逐步实施老城区保护工程，开展湖北会馆等30幢古民居、古作坊、古会馆建筑的维修保护工作；进一步加强对湖田民窑遗址和古瓷矿、古窑业、古水碓遗址的保护工作。科学规划建设和管理城市。

第一，加强城市规划。要重点规划以御窑为核心的历史文化保护街区和以昌南大道及昌南湖为中心的生态新区，突出抓好西瓜洲韭菜园地区、景德大道东段地区、景东大道片区、陶瓷文化创意产业园区、高新产业开发区三期等五个片区的规划编制、控制性详规编制及景观设计工作。

第二，全面实施新一轮土地利用总体规划。修编完善小城镇建设中长期规划‘和示范镇总体规划，全面启动建制镇控规。

第三，加强城市重大基础设施建设。要保障电力能源，推进电厂“上大压小”工程建设，力争第二台机组尽快并网发电，建设500KV景德镇变电站、220KV南山变电站；加快天然气管网建设，推进天然气利用工程；完善大交通格局，尽快开工九景衢铁路、皖赣铁路电气化改造工程，加快德昌、祁浮高速景德镇段等重大项目建设，加大国省干线公路改造和县乡公路管养力度；稳定和增加航班数量，积极开辟新的航线；争取开工建设浯溪口水利枢纽工程。

第四，认真清理和调整在建项目。

要缩短建设战线，在建项目要分轻重缓急，集中财力用于建设和完善重点防洪排涝体系、污水处理系统和棚户区改造；要缩短建设工期，基本完工项目要尽快收尾，投入使用。

第五，强化城市管理。加大市容环境整治和综合执法力度，加强城管执法队伍建设，改革城管执法体制，组建城管综合执法机构，严厉打击违法用地和乱搭乱建行为。要推行数字城管，完善“两级政府、三级管理、四级网络”的环卫管理体系。

第六，深入开展创建工作。提高城市公共文明指数，争创全国文明城市、国家卫生城市、国家环保模范城市和国家生态园林城市。加强城市生态和环境建设。以“一大四小”工程为重点做好造林绿化工作，重点抓好景鹰高速、杭瑞高速、九景高速洪源段、206国道、景瑶线、乐德线等通道绿化，抓紧城市及周边森林公园和湿地公园建设，加大森林和林地保护力度，完成造林8.73万亩；实施节能减排工程，加大对高耗能、高污染行业治理力度，不断降低单位GDP能耗和主要污染物排放总量；加快实施再生资源回收体系和再生资源循环利用体系建设；着力推进节水型社会建设，开展水生态环境治理，加快推进全年饮用国家一类标准水工程。统筹发展县域经济。要按照经济发展总体规划，把县域经济摆到经济发展大局中谋划，充分发挥各县（市、区）的比较优势，加快区域特色优势产业发展。乐平市应注重依托较好的工业基础，重点发展医药、食品、建材等产业，大力发展现代农业，建设“江南菜乡”。浮梁县应依托良好的生态资源，加快发展旅游、生态农业和绿色食品。昌江区应发挥城乡结合部优势，重点发展战略性新兴产业配套产业和现代服务业。珠山区应重点发展房地产业和商贸物流、商务会展、文化娱乐等现代服务业。在发展县域经济的同时，立足产业发展，走多样化的城镇化道路，重点建设工业型、旅游型、工矿型、城郊型、商贸及农副产品加工型五类城镇，统筹示范镇带动小城镇发展。

（四）依托文化资源优势，加快旅游产业发展

全面提升瓷都旅游产业的整体水平，逐步把我市建设成为旅游集散中心、国际旅游高地、陶瓷购物天堂和绝特历史文化与自然生态之旅的最佳目的地。2011年，力争全市接待国内外游客超过1500万人次，旅游总收入超过80亿元。提升景区档次和品位。启动古窑·民俗博览区和高岭·瑶里创建国家AAAAA级旅游景区活动，完善浮梁县古县衙、洪岩仙境等重点旅游景区的功能，加快建设乐平市江南蔬菜旅游休闲体验区，大力创建一批A级乡村旅游点。加大旅游推介力度。按照瓷源浮梁、古镇珠山、绿色昌江、多彩乐平的定位，树立区域旅游品牌形象，办好瓷博会、瓷文化旅游节和茶文化旅游节，举办中国产瓷区陶瓷文化旅游嘉年华活动，借助新闻媒体和国内外知名人士开展旅游推介，积极扩大港澳台地区、亚洲和欧洲的客源，加强“六山两湖”区域合作，打造景德镇一婺源一黄山、景德镇一庐山一三清山等精品旅游线路。强化旅游服务功能。规划建设游客服务中心、旅游交通服务中心、游客集散中心；推动现有星级酒店“加星、扩容、升级”，提高接待能力和水平，实现五星级饭店零的突破；加强旅游人才的教育和培训工作，培养和造就一支高素质的导游队伍；鼓励研发具有地方特色的旅游商品，扩大旅游购物消费比重。

（五）坚定不移推进改革，不断提高对外开放水平

稳步推进各项改革。完成国有农垦企业改革，继续做好已基本完成改革的国有企业遗留问题处理工作；着力抓好基本医疗保障制度、基层医疗卫生服务体系、基本公共卫生服务均等化等重点改革任务，努力解决群众“看病难、看病贵”问题；完善财税管理体制，加强地方性债务控制和管理，建立规范使用专项资金的长效机制，规范个人所得税征管秩序，建立和完善高收入群体的收入申报制度，加强艺术品交易的个人所得税征管；支持陶瓷股份公司、世龙实业等企业上市融资，积极培育后备上市资源，鼓励和引导民间资本投资基础设施、战略性新兴产业和社会公共事业；进一步加快信用担保体系建设，实现两家以上小额贷款公司开业；开展兴乡强镇改革试点，稳定和完善农村基本经营制度，推进林权制度配套改革。同时，大力发展非公有制经济，激发全民创业的积极性和创造性。坚持大开放主战略。第一，提高招商引资质量和水平。进一步加强与世界名优企业、中央企业、上市公司和知名民营企业的对接，更加注重引进拉动力强、带动性大、附加值高和环境污染小的重大项目。创新招商方式，借助重大招商活动，深入开展小分队招商、产业招商、以商招商。第二，主攻园区平台建设。要突出园区特色，构建产业配套体系，提升项目承载能力，提高产业聚集程度，优化园区投入产出比。第三，推动对外贸易稳步发展。要积极开拓新兴出口市场，大力发展加工贸易，着力优化出口结构，力争出口额超千万美元的出口企业达到15家。

（六）着力改善民生，统筹推进各项社会事业

大力实施民生工程。2011年，市本级财政安排1.82亿元，重点用于支持就业创业、社保、教育、医疗和住房等民生事业的发展。要把高校毕业生、农村转移劳动力、城镇就业困难人员的就业问题作为工作重点，千方百计增加就业。零就业家庭就业安置率实现100%，新增转移农村劳动力达1.87万人，劳动力转移培训达1.62万人，其中创业培训0.23万人；要扩大社会保险覆盖面，城镇基本养老保险参保缴费达22.71万人，基金征缴总额6.42亿元，被征地农民参加养老保险达3.2万人，巩固新型农村社会养老保险试点成果，参保人数达11.8万人，失业保险基金征缴达2680万元，城镇职工工伤保险参保达24.3万人，农民工参加工伤保险达6.6万人，城镇职工和居民基本医疗保险参保49.06万人，城镇居民基本医疗保险覆盖率达到100%，完善新型农村合作医疗制度，统筹基金使用率85%以上；新建廉租住房2840套、公共租赁房400套，改造棚户区16万多平方米，为农村困难群众新建、重建住房129户，维修住房46户；推进扶贫开发，移民扶贫搬迁430人，整体推进重点扶贫村18个。同时，全面完成省政府下达的其他各项民生工程目标任务。协调发展社会事业。

第一，坚持把教育摆在优先发展的战略地位。要统筹城乡资源，积极发展学前教育，优化中小学布局，均衡发展义务教育，继续支持景德镇高等专科学校的“建院升本”工作。改善农村学校和薄弱学校办学条件，大力发展职业教育，协调发展特殊教育、继续教育和老年教育。

第二，推进科技进步。以建设国家创新型城市为抓手，建立健全科技创新机制体制，搭建科技创新平台，打造充满生机和活力的创新型城市。

第三，促进文化事业繁荣发展。继续做好非物质文化遗产申报工作，加强文物保护，积极稳妥做好有线电视数字化整体平移工作。

第四，加强重大疾病预防控制，健全农村医疗服务网络，大力发展社区卫生服务，完善覆盖城乡居民的食品药品安全保障体系。第五，加强体育设施建设，改善全民健身条件。第六，稳定低生育水平，综合治理出生人口性别比偏高问题，提高出生人口素质。积极应对人口老龄化，扶持老龄事业发展。提高社会管理水平。以社会矛盾化解、社会管理创新、公正廉洁执法三项重点工作为抓手，切实维护社会稳定。健全社区管理服务体系，提高社区管理能力；高度重视信访稳定工作，进一步完善矛盾纠纷调处机制，尽快启用市人民来访接待中心；加强应急管理工作，不断提高处置各类公共突发事件的能力和水平；加强对自然灾害的预警预测，抓好防汛抗旱、森林防火、重大动植物防疫等工作，深入治理矿山地质环境；切实抓好安全生产，深入推进重点行业领域安全生产专项整治和隐患排查治理，坚决遏制重特大安全事故的发生；加强社会治安综合治理，完善社会治安防控体系建设，增强人民群众安全感；扩大法律援助范围，降低法律援助门槛；广泛开展法制宣传教育，启动“六五”普法规划；加强国家安全工作，推进武警部队现代化建设和国防后备力量、民兵预备役部队建设；加强“双拥”工作，努力实现全国双拥模范城“五连冠”。

（七）进一步加强自身建设，提高政府工作效能

坚持依法行政，推进科学民主决策。把依法行政贯穿到政府工作的各个领域、各个环节，严格按照法定程序、法定权限、法定职责行使人民赋予的权力。坚持依法科学民主决策，要加强行政决策程序建设，健全政府重大事项决策规则，完善公众参与、专家咨询、合法性审查、集体讨论决定的决策机制，推进决策科学化、民主化、制度化；严格规范公正文明执法，要健全行政执法程序，规范执法检查活动，依法依规有效化解社会矛盾纠纷；深入推进政务公开，要完善政府新闻发布、政府信息公开和各类公开办事等制度，努力打造阳光政务；完善行政监督机制，要依法接受人民代表大会及其常委会的法律监督和人民政协的民主监督，及时办理人大代表建议和政协委员提案，认真听取各民主党派、工商联、无党派人士、人民团体和政府参事的意见，强化监察、审计等专门监督，高度重视人民群众监督和新闻舆论监督。改进工作作风，提升服务水平。

大兴调查研究之风，尊重群众首创精神，问政于民、问计于民、问需于民；坚持实事求是，知实情、谋实招、办实事、求实效，努力建设务实型机关；加快转变政府职能，进一步推进行政审批“两集中，两到位”，清理规范行政审批事项，逐步推进网上审批和电子监察；加强公共资源交易中心建设，完善行政服务体系；强化督查机制，加强跟踪落实和监督检查，确保各项重点工作高效、有序地开展；以扎实开展“发展提升年”活动为抓手，进一步提升机关办事效能和服务水平。加强自身建设，坚持廉洁从政。要坚持标本兼治、综合治理、惩防并举、注重预防的方针，加强以完善惩治和预防腐败体系为重点的反腐倡廉建设。要逐步建立健全公共权力阳光运行和监控体系、从严惩治腐败体系、群众利益维护体系、反腐倡廉教育体系、反腐倡廉制度和执行评估体系；要深入推进专项治理工作，继续深化工程建设领域突出问题和“小金库”专项治理，切实抓好医药购销和医疗服务中突出问题专项治理，深入开展庆典、论坛过多过滥和公务用车间题专项治理，着力解决反腐倡廉建设中人民群众反映强烈的突出问题；要加强对国有企业“三重一大”决策制度执行情况的监督检查；强化领导干部经济责任审计和投资工程审计；要认真落实中央有关厉行节约的规定，牢固树立过紧日子的思想，坚决反对铺张浪费和奢靡之风，从严控制一般性支出，大力削减行政成本，把有限的财力更多用在科学发展和改善民生上。

各位代表，2011年是“十二五”规划的开局之年，做好今年的各项工作，意义重大，使命光荣！让我们高举中国特色社会主义伟大旗帜，更加紧密地团结在以胡锦涛同志为总书记的党中央周围，全面贯彻落实科学发展观，在省委、省政府和市委的领导下，进一步解放思想，坚定信心，团结拼搏，排难前进，努力实现“十二五”发展的良好开局，为瓷都科学发展、进位赶超、绿色崛起打下坚实基础！
